# Out of the box: toward new frameworks for understanding human microbiomes

**DOI:** 10.1128/msphere.01000-24

**Published:** 2026-01-12

**Authors:** Ariangela J. Kozik

**Affiliations:** 1 Department of Molecular, Cellular, and Developmental Biology, University of Michigan 1259, Ann Arbor, Michigan, USA; 2 Department of Internal Medicine—Division of Pulmonary and Critical Care Medicine, University of Michigan 1259, Ann Arbor, Michigan, USA; The University of Arizona, Tucson, Arizona, USA

**Keywords:** human microbiome, ethics, interdisciplinary

## Abstract

The study of the human microbiome (mirroring broader practice across biomedical science) has historically defaulted to the use of simplified, socially constructed "boxes,” such as racial and ethnic labels, that fail to accurately capture human variation and fundamentally misdirect the search for mechanisms to explain differences in health outcomes. Five years ago, I proposed a “frameshift,” a fundamental conceptual shift away from relying on these categories and toward a more nuanced, careful approach to the complexity of human variation. Moving “out of the box” means tackling the difficult but essential work of analyzing microbial variation through a systems lens, connecting large-scale ecosocial drivers to individual mechanisms and outcomes. In this Full Circle review, I discuss rapid progress in the field toward this new framework and argue that by adopting transdisciplinary methods, we can generate more accurate, actionable, and equitable solutions for human health.

## INTRODUCTION

Microbiome research is revolutionizing the way we understand human health, offering new insights that impact everything from how our cells and microbial cells interact to global health trends. Five years ago, I shared my vision for a “frameshift” in human microbiome research (1). The core idea was to stop treating a socially defined “race” as a biological cause for disease and instead focus on the social and environmental factors that truly drive differences in health outcomes (e.g., poverty, stress, insufficient access to care, etc.). This shift is now more vital than ever as contemporary societal movements challenge how science is communicated and conducted. Including social context in the study of humans and our microbiomes will enhance our understanding of the relationship between microbiomes and health outcomes. Since 2020, several investigators have expanded the field’s research scope, highlighting the problems with using oversimplified labels, especially racial and ethnic terms, in microbiome research. My work, alongside others, is extending the field’s boundaries by advocating for a more careful understanding of these social contexts and their implications for biology. To improve the validity and impact of our findings, it is essential to use methods that acknowledge the rich tapestry of human diversity beyond these reductive racial labels.

## THE PROBLEM WITH PROXIES

The core proposition of the prior article was to rethink how race and social factors are used and discussed in biomedical research (1). In the United States and other contexts, the socially defined category of “race” often acts as a stand-in for health-determining social factors (2). The misuse of race in scientific studies often leads to misleading conclusions that overlook the biological irrelevance of race as a causal factor (detailed discussions can be found elsewhere [3–5]). Instead of viewing social exposures and environmental factors as mere “confounders” to be controlled, they should be considered measurable causes of health and disease processes. When race is used as a potentially causal factor, these more significant and actionable drivers are often neglected. However, if we rigorously collect and integrate additional, more nuanced data, researchers will generate findings that can lead to interventions rather than reinforcing flawed “biological” concepts of race.

## FROM CONCEPT TO COLLABORATION

Echoing and expanding on the call from the frameshift article, a now frequently cited 2021 *mSphere* article “Chasing ghosts: race, racism, and the future of microbiome research” by De Wolfe et al. (6), discussed in detail the problematic use of race as a biological proxy. The authors defined “ghost variables” as vague terms that imply racial context without explicitly naming the actual drivers of biology. They advocated for a more accurate focus on associative factors that can affect microbiome change, such as sanitation access, exposure to pollution, and dietary differences. They also brought specific attention to structural drivers—the sociocultural, economic, political, and historical structures (like redlining or colonialism) that underlie society but are often ignored in microbiome research design, execution, analysis, and discussion.

Simultaneously, the Microbes and Social Equity (MSE) Working Group was established in 2019 at the University of Maine to promote interdisciplinary research that bridges microbiology, sociology, and bioethics (7). This international collective of scholars argues that humans have vastly different experiences of health, resources, and environment. MSE’s goal is to develop collaborative transdisciplinary teams to understand the interplay of microorganisms, individuals, societies, and ecosystems. If our desired future is one where good health is within grasp for all our neighbors, MSE argues that research must integrate both biological and social sciences. Since its inception, MSE has led annual virtual symposia (8) that bring together experts across many research areas, disciplines, and training stages. These symposia remain impactful spaces for transdisciplinary engagement and conversation.

This approach can lead to more informative findings and interpretations, as was shown in a key re-analysis (9) of Human Microbiome Project data. This study used the social vulnerability index (SVI) to re-examine previously published racial/ethnic differences in microbiome diversity. The SVI is a Centers for Disease Control and Prevention (CDC)-validated measure of factors that contribute to social vulnerability (10). The index considers not only socioeconomic status, but also factors, such as household composition, the presence of disability, available transportation, and language barriers. The investigators found that the SVI was associated with differences in microbial diversity across body site and may explain the previous attribution of these differences to race/ethnicity (9). This work supports the argument that social vulnerability assessments or similar measurements of social and environmental factors are critical in human microbiome research, demonstrating the potential for transdisciplinary collaboration to generate insights into health disparities.

Another excellent example of the field’s progress in this area is the Isala Sisterhood Project. This project is a global, citizen science initiative that seeks to resolve the lack of available microbiome data that represent the global population (11). Starting in Belgium and expanding globally across continents, this project uses a public engagement approach to map the vaginal microbiome in self-reported healthy women. The Isala Sisterhood is crucial because it addresses the Western-centric bias in microbiome research, where traditional classifications of “healthy” and “dysbiotic” vaginal microbiomes are based on work from a very small slice of the global population. By recruiting thousands of participants across continents, the project is revealing that a significant percentage of women do not fit neatly into these predefined, simplistic “type” categories (12). This work provides evidence as to the power of the core argument of the frameshift piece: human microbial diversity transcends our existing, limiting categories. The Isala and sister projects show that increasing geographical, social, and cultural representation is essential to developing precise and globally relevant diagnostic and therapeutic strategies for women’s health.

## CONSENSUS ACROSS DISCIPLINES

Fostering interdisciplinary dialogue on this topic is crucial and can move the broader scientific community forward. In 2023, the National Academies of Sciences, Engineering, and Medicine (NASEM) released the report “Using population descriptors in genetics and genomics research” (13). While the report focuses on genetics and genomics, it has clear implications for microbiome science. The report (developed by a team of experts in genetics, sociology, law, biological informatics, and genomics) examines the historical and current use of these descriptors, their challenges, and limitations while offering key recommendations for change. The report calls for moving away from simplified, racialized proxy labels and categories in favor of more accurate population or environmental descriptors. Moreover, the report highlights the common confusion between concepts like race, ethnicity, and ancestry, which can result in flawed interpretations that support biological determinism or essentialism. It includes guidance on identifying social determinants of health and defining social and environmental factors, as well as assessing the appropriate use of race and ethnicity in specific research questions. The report is comprehensive and provides a common vocabulary for future research and decision-making, even in contexts outside of genetics and genomics not directly addressed in the report. The authors also situate their work firmly within the guiding principles that underlie modern scientific pursuit and ethical responsibility—“for scientific research to be…ethically conducted and empirically valid.”

Recognizing the urgent need to translate these guidelines for our discipline, my MSE colleagues and I published “Prioritizing precision: guidelines for the better use of population descriptors in human microbiome research” in *mSystems* (14). Our paper affirmed the NASEM report’s key guidance: moving away from simplified proxy labels and discussed specifically what this means for microbiome research. We also included an in-depth summary of the history of race/ethnicity/ancestry labels and categories as used in the United States and discussed the associated problems and considerations that arise when inappropriately applied to microbiome data. We also provide actionable guidelines for microbiome researchers to use in future work, prioritizing three main themes. First, clearly define terms, stating the definition and justification for using any population descriptor; second, avoid re-biologizing race by refraining from presenting or interpreting microbial differences as a biological trait that can be directly attributed to a racial group; and third, move toward mechanism by prioritizing the collection of measurable social and environmental factors to understand why microbial differences may exist. By providing these guidelines, we hope to ensure that the progress made in genomics through the NASEM report is immediately translated to the microbiome sciences.

These discussions have reached professional societies as well. In a recent American Thoracic Society (ATS) workshop report, my colleagues and I performed a deep literature review on the persistent racial disparities in pediatric asthma in the United States (15). In a literal “Full Circle” moment, I was able to work on this report with the senior author of the “time’s up” editorial (16) that I discussed in my original *mSphere* “frameshift” article. The editorial discussed the results of a 2019 study from the National Heart, Lung, and Blood Institute’s Severe Asthma Research Program, which found that accounting for community, socioeconomic, and environmental exposure variables eliminated previously reported and well-known disparities in asthma-related emergency room visits among Black Americans that had been attributed to “race” (17).

The ATS report provides several additional examples of how socially-defined race alone cannot explain the differences in pediatric asthma outcomes. We instead propose approaches to study this problem in the context of systemic factors (social caste, environmental exposures, access to care, etc.). A key concept we define is racialization, the historical process by which visible features of humans (phenotypes) were collapsed into discrete, supposedly “biologically meaningful” racial categories. We then discuss further how these categories are social and not accurate or precise representations of meaningful biological variation of our species that can be directly linked to health outcomes. We argue that they are, at best, oversimplified proxies. Our revised framework incorporates socioeconomic and sociopolitical contexts, social caste (factors like occupation, education, wealth, status), and healthcare access to paint a more complete picture of the factors influencing pediatric asthma outcomes. This approach supports more comprehensive studies and analyses and community-engaged research and will ultimately result in targeted and effective interventions, advancing equitable healthcare for children with asthma.

## METHODOLOGICAL CHALLENGES AND OPPORTUNITIES

The call for a frameshift is not just conceptual; it demands new approaches to research methodology and data collection. Moving beyond relying on race as a proxy variable requires collecting rich, complex data that capture the lived experience of study participants that is relevant to our questions. These present both challenges and substantial opportunities for innovation. For example, we must pivot from simple demographic questionnaires to integrating validated instruments to quantify environmental exposures, psychosocial stressors, and socioeconomic factors. Robust transdisciplinary collaboration is essential here. Social scientists, anthropologists, and bioethicists will be crucial in helping microbiologists select appropriate, nuanced variables and ensure data collection is done ethically and contextually. The goal is to build microbiome study metadata that are “socially aware,” meaning that they explicitly link microbial variation to upstream structural, social, and environmental determinants, thereby offering clearer targets for interventions.

## BENEFITS BEYOND ETHICS

While the ethical imperative to abandon the use of flawed proxies is clear, the scientific justification is even more profound. Relying on ill-defined, socially constructed variables like race to explain biological phenomena limits our potential for discovery. When studies are anchored to and informed by disproven ideas about the connection between race and biology, the resultant interventions are often incomplete or ineffective. If we instead work together across disciplines to innovate and expand our methods and embrace the complexities of human biology, we will generate data that are empirically stronger and more reproducible across human populations. Ultimately, this will lead to the discovery of mechanisms, treatments, and interventions that are precise, globally relevant, and propel us toward new horizons for human health.

In today’s rapidly evolving landscape, as microbial science—and science as a whole—navigates significant uncertainties and pressures to restructure our relationship to information, evidence, and communication, we can and should anchor ourselves to evidence and truth. Human microbial and genetic diversity transcends our socially constructed categories. These categories can only ever partially describe us. Our collective health, innovation, and discoveries are enriched, not hindered, by embracing the heterogeneity vital to life on this planet. There is much work to do.
